# Seroprevalence of peste des petits ruminants and bluetongue in goat population of Meghalaya, India

**DOI:** 10.14202/vetworld.2018.1689-1691

**Published:** 2018-12-17

**Authors:** A. Karam, K. Puro, S. Das, I. Shakuntala, R. Sanjukta, A. A. P. Milton, S. Ghatak, A. Sen

**Affiliations:** 1 Animal Health Division, ICAR-RC for NEH Region, Umiam-793103, Meghalaya, India; 2 Mizoram Centre, ICAR-RC for NEH Region, Kolasib- 796081, Mizoram, India

**Keywords:** bluetongue, goat, peste des petits ruminants, seroprevalence, virus

## Abstract

**Aim:**

This study aimed to determine the seroprevalence of peste des petits ruminants (PPR) and bluetongue (BT) in goats’ population in the state of Meghalaya of Northeast India.

**Materials and Methods:**

The serosurveillance study was done from the random sampling (n=598) of blood collected from five districts (Ri-Bhoi, East Khasi Hills, West Khasi Hills, Jaintia Hills and West Garo Hills) of Meghalaya. The presence of antibodies against PPR and BT in the samples was detected by indirect enzyme-linked immunosorbent assay (ELISA) method for PPR and competitive ELISA for BT.

**Results:**

The results showed the overall seropositivity of PPR and BT at 7.19% and 60.20%, respectively. West Garo Hills recorded the highest seroprevalence of both PPR (9.81%) and BT (68%) and 3.6% of the samples tested positive for both PPR and BT.

**Conclusion:**

The random survey results indicating the presence of PPR and BT have specific implication in epidemiological perspectives since it highlights the prevalence under natural situations, where the subclinical, inapparent, or non-lethal or recovery of infection was suspected in unvaccinated animals. It also warrants further studies to suggest appropriate control measures to prevent the spread of infection.

## Introduction

Goat farming is gaining importance in the northeastern part of India mainly due to their short generation interval and a higher rate of prolificacy. It also possesses high efficiency in transforming feed into milk and meat and capable of selective browsing on the vegetation found in the region. Although goats are known to be relatively resistant to many infectious diseases, they are under constant threat of diseases such as peste des petits ruminants (PPR) and bluetongue (BT) which are the two major diseases of sheep and goat listed under category “A” of disease by OIE and are endemic in India [1,2]. PPR is characterized by mucopurulent nasal and ocular discharge, necrotizing and erosive stomatitis, enteritis, and pneumonia. It is caused by *Morbillivirus* belonging to family Paramyxoviridae. In India, the first PPR outbreak was recorded in Tamil Nadu in 1987 [3]. Since then, outbreaks of PPR has been reported from various parts of the country. BT is an insect-borne viral disease of ruminants characterized by inflammation of mucous membranes, congestion, swelling, and hemorrhages. The causative agent is BT virus of the genus *Orbivirus* in the family Reoviridae [4]. The virus is transmitted between vertebrate hosts by *Culicoides* species [5]. The clinical disease and mortality are observed in sheep, especially in the southern states of India [6], and in northern India, the first outbreak in goat was reported from Dehradun [7]. Reports on studies of seroprevalence from various states have been documented [8] since serological investigation is considered a faster means of assessing the prevalence of infection.

The study on the prevalence of PPR in goats in northeast India recorded 11.63% positive from random samples [9] which gave some insights into the evidence of the presence of PPR in the region. Besides, we also reported the high mortality of goat from an outbreak of PPR [10] in Meghalaya, but there are very few reports on the status of BT disease in Meghalaya.

Therefore, this serosurveillance study was done to determine the prevalence and understand the status of PPR and BT in the goat’s population in the state of Meghalaya.

## Materials and Methods

### Ethical approval

As per CPCSEA guidelines, studies involving samples for disease surveillance do not require the approval of Institutional Animal Ethics Committee. However, samples were collected as per standard sample collection methods without any harm or stress to the animals.

### Collection of sera samples

The sampling was done for a period of 6 months during August 2017–February 2018. Serum samples of unknown antibody status (n=598 goats) of non-vaccinated goats were collected as per the random sampling frame designed by National Institute of Veterinary Epidemiology and Disease Informatics, Bengaluru, from various villages in the selected districts of Meghalaya. During the period, caprine sera samples mostly of Assam Hill goat and some non-descript were collected from five districts, that is, Ri-Bhoi (n=37), East Khasi Hills (n=110), West Khasi Hills (n=132), Jaintia Hills (n=44), and West Garo Hills (n=275). The samples were stored in deep freezer (−20°C) until screening was done.

### Screening of the samples

The screening for the presence of PPR and BT antibodies was done using commercial enzyme-linked immunosorbent assay (ELISA) kit according to manufacturer’s instructions. The anti-PPRV nucleoprotein antibodies in serum were detected using indirect ELISA kit (ID screen^®^ PPR competition, IDvet Genetics, Grabels, France). The result was calculated as sample positivity (S/N) for antibodies in percentage. The sample is considered as positive if the S/N is ≤ 50% or doubtful if it is between 50-60% and negative if ≥ 60%. The antibody against bluetongue viruses was detected using IDEXX BT Competition Ab Test (IDEXX Laboratories Inc., Westbrook, Maine, US). The result was calculated as sample positive for antibodies in percentage by determining the cutoff value (mean OD of positive control*0.15). The sample is considered positive if the OD of the sample is less than or equal to cutoff value or negative if the OD is greater than cutoff value.

## Results and Discussion

Meghalaya is the northeastern state of India located in the eastern Himalaya surrounded by Assam in the east, north, and west and Bangladesh in the south. The topography, climate, and socioeconomic conditions make the people depend more on animal husbandry activities. The practice of traditional agriculture in hilly areas allows only about 10% of the total land in the state for farming. Therefore, rearing of small ruminant seems to be a viable option for livelihood sustenance. The population of goats in Meghalaya as per the Livestock Census, 2012, is 4.72 lakhs with an increased growth rate of 29.23% compared to 2007 census (http:/megahvt.gov.in/livestockcensus.html). However, mortality due to diseases affected the production. The results of seroprevalence of PPR and BT among goats in five districts of Meghalaya are summarized in Table-1. In the present study, the overall seropositivity of PPR and BT was 7.19% and 60.20%, respectively, with 43 samples positive for PPR and 360 samples positive for BT from the total of 598 samples. The highest seropositivity of PPR (9.81%) was detected in samples from West Garo Hills followed by West Khasi Hills (6.81%), Ri-Bhoi (5.40%), Jaintia Hills (4.54%), and East Khasi Hills (2.72%). For BT, the highest seropositivity of 68% was recorded from West Garo Hills followed by Ri-Bhoi (62.16%), Jaintia Hills (61.36%), East Khasi Hills (37.27%), and West Khasi Hills (36.36%). In the study, it was found that 3.6% of the samples from West Garo Hills were positive for both PPR and BT.

**Table-1:** Percent seropositivity of PPR and BT in five districts of Meghalaya.

District	PPR (%)	BT (%)	PPR+BT (%)
Ri-Bhoi	5.40 (2/37)	62.16 (23/37)	−
East Khasi Hills	2.72 (3/110)	37.27 (41/110)	−
West Khasi Hills	6.81 (9/132)	36.36 (48/132)	−
Jaintia Hills	4.54 (2/44)	61.36 (27/44)	−
West Garo Hills	9.81 (27/275)	68 (187/275)	3.6 (10/275)
Total	7.19 (43/598)	60.20 (360/598)	1.6 (10/598)

−=Negative, PPR=Peste des petits ruminants, BT=Bluetongue

The seroprevalence of PPR in goats of northeast India was reported to be 11.63% [9] while the recent report of 13.18% in goats of Assam [11]. In the present study, the recorded positivity ranging from 2.72% to 9.81% indicated the presence of the PPR in all five districts of Meghalaya. The seroprevalence rate of BT in goats in the present study of Meghalaya was 60.20% with variation in different district ranging from 36.36% in West Khasi Hills to 68% in West Garo Hills. The seroprevalence of BT in goats reported by various workers [12] detected 43.56% BT in goat while 47% positivity was observed in the goats of coastal saline (Sunderban) areas of West Bengal [13] and 31.79% in the state of Assam [14]. The present finding of 60.20% is higher as compared to other reports. Although the incidence of BT in animals of Meghalaya is not reported so far, the present seroprevalence status indicates the presence of inapparent/subclinical or recovered from infections among the goat’s population in the state for the 1^st^ time. It is also to be noted that 3.6% of the samples from West Garo Hills tested positive for both PPR and BT. Migratory flocks of nomads have been thought to be a source of the infection of BT [15] and PPR [16] in new areas. In this case, the movement of animal from the neighboring state of Assam is thought to be a crucial factor for maintaining the high seropositive animals in the state since there is interstate live animal market which caters to the buying and selling of livestock’s including goats. An outbreak of PPR in goats in Ri-Bhoi district of the Meghalaya was attributed to the transportation of goats from Assam [10]. Furthermore, the environmental stress, particularly hot and humid climate or low environmental temperature bolstered by humidity and nutritional deficiency contributes to the precipitation of these diseases [17]. The climatic conditions of the West Garo Hills are characterized by hot and humid rainy seasons during summers with high average annual rainfall. This area recorded higher seroprevalence of PPR and BT than other districts of Meghalaya. The density of the animal population may also play a key factor in the spread of the disease since West Garo Hills have higher numbers of goat’s population as compared to other districts having sparse animal population, and we observed some animals were seropositive for both PPR and BT.

## Conclusion

From the present study, it is indicated that both PPR and BT are prevalent in goats throughout the state. Therefore, a holistic study by including other ruminants such as cattle and sheep are required to determine the seroprevalence as well as characterize the viral isolates from clinical cases for effective surveillance and establishing control strategies like vaccination for these important animal diseases in ruminants. At present, there is no vaccination of sheep and goats undertaken in NE region. The northeastern border states of India share international boundaries with Bangladesh, Nepal, Myanmar, China, and Bhutan. Therefore, the transboundary migration of animals must also be monitored for proper management of the disease.

## Authors’ Contributions

The work mandate and sampling plan were communicated to IS and AS for investigation. They planned and tasked the work to others. AK, SD, AAPM, and RS went for field investigation and collection of samples from various districts. AK, KP, and SG did the serological work. AK and KP drafted and revised the manuscript. All authors read and approved the final manuscript.
